# Supplementary material for: Acceptance and hesitancy of COVID-19 vaccine among Nepalese population: A cross-sectional study
Source: PLoS One. 2022 Oct 20;17(10):e0275922. doi: 10.1371/journal.pone.0275922 (PMC9584529; doi:10.1371/journal.pone.0275922)
Supplement: S1 File — (DOCX) [file pone.0275922.s001.docx]

## Questionnaire:

**Section A**

1. Age:
2. Gender:
3. Address:
4. Education Level:
5. Occupation:

**Section B: Vaccinated Status**

1. Have you been vaccinated with COVID 19 vaccine?
2. Yes
3. No
4. Don't Know

**Section C**

1. Will you get COVID-19 vaccine if made available?

a. Yes

b. No

c. Not sure

If you want to take vaccine, why would you want to get it? Put a tick mark on your opinion.

- 1. I want to protect myself from having COVID-19 in the future.

1. Completely agree
2. Agree
3. Disagree
4. Completely Disagree
   1. Vaccination decreases my chance of getting COVID-19 or its complications.
5. Completely agree
6. Agree
7. Disagree
8. Completely Disagree
   1. I want to protect my family/ members of my household against having COVID-19 in the future.
9. Completely agree
10. Agree
11. Disagree
12. Completely Disagree
    1. I want to protect my community against having COVID-19 in the future.
13. Completely agree
14. Agree
15. Disagree
16. Completely Disagree
    1. Vaccination would ease the precautionary measures including lock down, quarantine, and travel ban and make daily life normal.
17. Completely agree
18. Agree
19. Disagree
20. Completely Disagree

If you don’t want to get vaccinated, then why would you not get vaccinated? Put tick on your opinion.

**Section D**

- 1. I am afraid of the queue present in the vaccination center.

1. Completely agree
2. Agree
3. Disagree
4. Completely Disagree
   1. I believe that natural immunity is sufficient, and I don’t think I need to take the vaccine.
5. Completely agree
6. Agree
7. Disagree
8. Completely Disagree

8.3 I am afraid of adverse effects of COVID-19 vaccine.

1. Completely agree
2. Agree
3. Disagree
4. Completely Disagree

8.4 I’m not sure of the effectiveness of the vaccination.

1. Completely agree
2. Agree
3. Disagree
4. Completely Disagree

8.5 COVID-19 infection is not serious so I don’t think I should take the vaccine

1. Completely agree
2. Agree
3. Disagree
4. Completely Disagree

8.6 I won’t have time to get vaccinated.

1. Completely agree
2. Agree
3. Disagree
4. Completely Disagree

8.7 I don't have enough information regarding COVID 19 Vaccine.

1. Completely agree
2. Agree
3. Disagree
4. Completely Disagree

**प्रश्नावलीः**

**खण्डकः**

१. उमेरः

२. लिंगः

३. ठेगानाः

४. शैक्षिकस्तरः

५. पेशाः

**खण्डखः**

६. के तपाईँले कोभिड-१९ विरुद्धको खोप लगाउनुभएको छ?

क) लगाएको छु

ख) लगाएको छैन

ग) थाहा छैन

**खण्डगः**

७. के तपाईँले कोभिड-१९ विरुद्धको खोप लगाउन पाइएमा लगाउनु हुनेछ?

क) लगाउँछु

ख) लगाउँदिन

ग) थाहाछैन

यदि तपाईं लगाउन चाहनुहुन्छ भने किन लगाउन चाहनुहुन्छ? आफ्नो बिचारमा चिन्ह लगानुहोस्।

७(१) म आफुलाईँ भविष्यमा कोभिड-१९हुनबाट बचाउन चाहन्छु।

क) पूर्ण सहमत छु

ख) सहमत छु

ग) असहमत छु

घ) पूर्ण असहमत छु

७(२) यस खोपले कोभिड-१९को संक्रमण हुने सम्भावना वा यसको जटिलता कम गराउँछ।

क) पूर्ण सहमत छु

ख) सहमत छु

ग) असहमत छु

घ) पूर्ण असहमत छु

७(३) म मेरो परिवार/घरका सदस्यहरुलाई भविष्यमा कोभिड-१९को संक्रमण हुनबाट बचाउन चाहन्छु।

क) पूर्ण सहमत छु

ख) सहमत छु

ग) असहमत छु

घ) पूर्ण असहमत छु

७(४) म मेरो समाजलाई भविष्यमा कोभिड-१९ को संक्रमण हुनबाट बचाउन चाहन्छु।

क) पूर्ण सहमतछु

ख) सहमत छु

ग) असहमत छु

घ) पूर्ण असहमत छु

७(५) कोभिड-१९ विरुद्धको खोपले सावधानीका उपायहरु जस्तै लकडाउन, क्वारेनटाईन र आवतजावतमा रोकहरुलाई कम गर्दै साधारण जिवन अवस्थामा फर्कन मद्दत गर्छ।

क) पूर्ण सहमत छु

ख) सहमत छु

ग) असहमत छु

घ) पूर्ण असहमत छु

**खण्ड घ**

८यदि खोप लगाउनुहुन्नभने किन लगाउनुहुन्न? आफ्नो बिचारमा चिन्ह लगाउनुहोस्।

८ (१) मलाई खोपकेन्द्रमा हुने लाइन देखी डर लाग्छ।

क) पूर्ण सहमत छु

ख) सहमत छु

ग) असहमत छु

घ) पूर्ण असहमत छु

८(२) मलाई हाम्रो प्राकृतिक रोगसँग लड्ने क्षमता पर्याप्त छ र खोपलगाउन आवस्यक छैन भन्ने लाग्छ।

क) पूर्ण सहमत छु

ख) सहमत छु

ग) असहमत छु

घ) पूर्ण असहमत छु

८(३) मलाई कोभिड-१९ विरुद्धको खोपबाट हुनसक्ने अज्ञात हानिकारक असरहरुदेखि डर लाग्छ ।

क) पूर्ण सहमत छु

ख) सहमत छु

ग) असहमत छु

घ) पूर्ण असहमत छु

८(४) म कोभिड-१९को प्रभावकारीता बारे विश्वस्त छैन।

क) पूर्ण सहमत छु

ख) सहमत छु

ग) असहमत छु

घ) पूर्ण असहमत छु

८(५) कोभिड-१९त्यति गम्भीर प्रकारको रोग होईन त्यसकारणले मलाई खोप आवस्यक छजस्तोलाग्दैन।

क) पूर्ण सहमत छु

ख) सहमत छु

ग) असहमत छु

घ) पूर्ण असहमत छु

८(६) मसँग खोप लगाउनकोलागी समय हुँदैन।

क) पूर्ण सहमत छु

ख) सहमत छु

ग) असहमत छु

घ) पूर्ण असहमत छु

८(७) मलाई कोभिड-१९ विरुद्धको खोपको बारे पर्याप्त जानकारी छैन।

क) पूर्ण सहमत छु

ख) सहमत छु

ग) असहमत छु

घ) पूर्ण असहमत छु
